# Energy-Adaptive Multi-Dimensional Learning Control for Federated Learning in Energy-Harvesting AIoT Systems

**DOI:** 10.3390/s26113522

**Published:** 2026-06-02

**Authors:** Dong Kun Noh, Changmin Kwak

**Affiliations:** 1 School of AI Convergence, Soongsil University, Seoul 06978, Republic of Korea; 2 School of AI Software, Soongsil University, Seoul 06978, Republic of Korea; byj010608@gmail.com

**Keywords:** federated learning, AIoT, energy-harvesting, energy-aware computing, model compression, adaptive learning, edge intelligence

## Abstract

This paper addresses the problem of efficient federated learning in energy-harvesting AIoT systems, where time-varying energy availability may lead to device blackouts and unstable learning performance. To address this issue, we propose an energy-adaptive multi-dimensional learning control framework that jointly determines model complexity and training intensity based on the real-time energy state of each device. This method integrates multiple control dimensions, including model pruning, quantization, knowledge distillation, and adaptive local training, into a unified decision mechanism under an energy constraint. Each device determines its participation in federated learning based on its residual energy relative to an energy threshold. When participating, the device selects a feasible learning configuration that jointly considers training intensity (e.g., epoch size and batch size) and lightweight learning operations to maximize learning effectiveness while preventing energy depletion. The proposed framework was implemented on a real-world testbed using NVIDIA Jetson Orin Nano devices under solar-energy-harvesting conditions. Our experimental results demonstrate that the proposed method significantly reduces device blackout while maintaining competitive model accuracy with respect to energy-unconstrained scenarios. These results highlight that joint control of multiple learning-cost factors is essential for achieving stable and efficient federated learning in energy-harvesting AIoT environments.

## 1. Introduction

With the rapid advancement of Internet of Things (IoT) technologies and artificial intelligence, AI-enabled IoT (AIoT) systems have emerged as a key paradigm for intelligent edge computing. In such environments, numerous distributed devices collaboratively collect and process data, enabling various applications, such as smart-environment creation, industrial monitoring, and autonomous-system development. However, the decentralized nature of AIoT systems introduces significant challenges in data management, communication efficiency, and privacy preservation.

Federated learning (FL) has recently attracted a significant amount of attention as an effective approach to addressing these challenges [1,2,3,4]. In FL, each device performs local training using its own data and shares only model updates with a central server, thereby avoiding the transmission of raw data. This decentralized learning paradigm not only preserves data privacy but also reduces communication overhead, making it particularly suitable for AIoT environments.

Meanwhile, there has been increasing interest in sustainable AIoT systems powered by renewable energy sources such as solar energy. Energy-harvesting (EH) AIoT devices can operate autonomously without relying on a fixed power infrastructure, making them attractive for long-term and large-scale deployments [5,6]. However, unlike conventional battery-powered systems, the available energy in EH-AIoT environments varies dynamically depending on environmental conditions such as solar intensity and weather [7]. This variability introduces critical challenges for federated learning, as insufficient energy may lead to device blackouts, interrupted training, and degraded model performance.

To address these issues, recent studies have explored energy-aware federated learning approaches [8,9,10]. These methods typically focus on controlling client participation based on resource availability, ensuring that only capable devices contribute to training [11]. Adaptive participation strategies have also been proposed, allowing devices to dynamically decide whether to participate in each training round based on their current energy state [12]. While these approaches improve system stability, they mainly treat energy awareness as a binary decision problem and do not fully utilize the available energy for optimizing the learning process itself.

In parallel, various model efficiency techniques have been proposed to reduce the computational and communication cost of federated learning. Model pruning removes redundant parameters to reduce model complexity [13,14], while quantization reduces numerical precision to improve computational efficiency [15,16]. In addition, approximate computing techniques have been explored to further improve energy efficiency in edge AI systems [17,18,19]. Knowledge distillation has also been widely adopted to transfer knowledge from large models to lightweight models, enabling efficient learning under resource constraints [20]. However, these techniques are typically applied independently and do not consider the dynamic and time-varying nature of energy availability in EH-AIoT environments.

Recent studies on energy-aware and energy-harvesting-aware federated learning have explored adaptive participation, resource scheduling, communication-efficient aggregation, transmission power control, and energy-aware training activation. These approaches have improved the feasibility of federated learning under resource and energy constraints by determining when, which, or how many clients should participate in each training round. However, most existing approaches mainly focus on client-level participation, scheduling, communication, or activation control. Once a client is selected, the local learning process is often performed with a fixed configuration or optimized only along a single dimension.

This observation leads to the main research gap addressed in this work. Existing energy-aware federated learning methods do not sufficiently explain how the internal learning configuration of each participating client should be adapted under time-varying harvested energy. In EH-AIoT systems, the learning cost of a client is determined not only by whether the client participates, but also by how much local training is performed, how complex the local model is, what numerical precision is used, and how much performance loss is recovered after model compression. Nevertheless, these control dimensions are usually considered separately in existing studies, even though they jointly affect both energy consumption and learning effectiveness.

To address this gap, this paper formulates energy-aware federated learning as a unified multi-dimensional learning-configuration control problem. The proposed framework does not claim novelty from the energy-threshold rule alone or from the individual use of pruning, quantization, and knowledge distillation. Instead, its main contribution is to use the available learning energy as a common constraint for jointly determining client participation, local training intensity, model pruning, quantization, and knowledge distillation within a single decision structure. Therefore, the proposed method extends energy awareness from binary participation or scheduling control to configuration-level learning control.

Based on this perspective, we propose an energy-adaptive multi-dimensional learning control framework for federated learning in energy-harvesting AIoT systems. Specifically, the proposed framework jointly regulates multiple aspects of learning cost, including model complexity, training precision, and learning intensity, based on the real-time energy state of each device. In the proposed framework, each device first determines its participation in federated learning based on its residual energy relative to an energy threshold. If participation is allowed, the device adaptively selects a feasible learning configuration that satisfies the available energy constraint. This configuration jointly considers training intensity, such as epoch size and batch size, and lightweight learning operations, such as pruning, quantization, and knowledge distillation. By doing so, each client can effectively utilize its available energy while avoiding excessive consumption that may lead to future instability.

**Figure 1:**
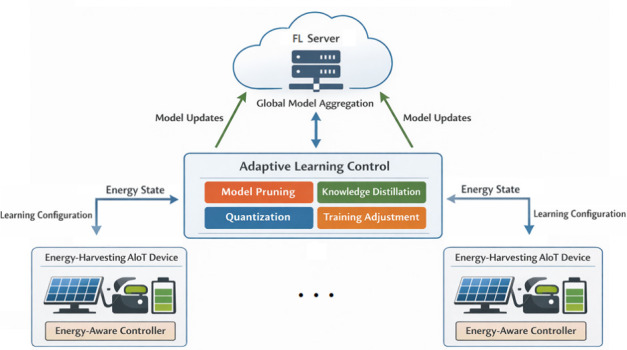
Overview of the proposed energy-adaptive multi-dimensional learning control framework. Each device dynamically determines its participation and learning configuration based on its energy state, while the central server aggregates model updates to construct a global model.

The overall concept of the proposed framework is illustrated in Figure 1. Each device monitors its energy state through an energy-aware controller and adaptively determines its learning configuration. The central server aggregates model updates from participating devices to construct a global model. Through this joint control mechanism, the proposed method enables efficient utilization of harvested energy while maintaining stable learning performance under dynamic energy conditions.

The main contributions of this paper are summarized as follows:

- We propose an energy-adaptive multi-dimensional learning control framework for federated learning in EH-AIoT systems, where the available learning energy is used to guide both participation and local learning-configuration decisions.
- The proposed framework jointly determines client participation, training intensity, and lightweight learning operations under a common energy constraint. In this process, the energy-threshold rule serves as a safety boundary for participation control, while pruning, quantization, and knowledge distillation are treated as controllable learning dimensions for configuring local training.
- We design a unified decision structure that integrates pruning, quantization, knowledge distillation, and adaptive local training into a single energy-adaptive learning process, enabling each client to dynamically select feasible learning configurations while balancing learning effectiveness and energy efficiency.
- We implement the proposed framework on a real-world AIoT testbed using NVIDIA Jetson Orin Nano devices under solar-energy-harvesting conditions, and we demonstrate that this method effectively reduces blackout while maintaining competitive learning performance under dynamic energy conditions.

## 2. Related Work

### 2.1. Energy-Harvesting AIoT Systems

Energy-harvesting AIoT (EH-AIoT) systems have been widely studied as a promising solution for enabling sustainable and long-term operation of distributed IoT devices [5,6]. By utilizing renewable energy sources such as solar, thermal, and vibration energy, EH-AIoT devices can operate without relying on a fixed power infrastructure. This characteristic makes them particularly suitable for large-scale and remote deployments.

However, unlike conventional battery-powered systems, the amount of harvested energy varies significantly over time depending on environmental conditions such as solar intensity, weather, and device location [7]. This variability introduces challenges in maintaining stable system operation, as devices may experience energy shortages or surpluses at different times.

To address this issue, previous studies focused on energy management strategies such as duty cycling, adaptive sensing, and energy-aware task scheduling [21]. These approaches are designed to ensure energy-neutral operation by balancing energy consumption and harvested energy. While effective for traditional IoT workloads, they are not directly applicable to AIoT systems that require continuous and computationally intensive learning processes.

Recent advances in energy-harvesting technologies further expand the potential of self-powered IoT and AIoT systems. For example, Cong et al. proposed a bio-inspired magnetically triggered snap-through mechanism that enhances mechanical-to-electrical energy conversion by applying impulsive excitation to triboelectric and piezoelectric energy-harvesting structures [22]. Their work demonstrates that advanced harvester designs can improve power output and support self-powered wireless sensing applications. However, such studies mainly focus on improving the energy-generation capacity of harvesting devices and validating sensing-level operation. They do not address how the harvested energy should be dynamically allocated to computation-intensive federated learning processes. In contrast, this work focuses on energy-adaptive learning control, where the harvested and residual energy of each AIoT device is used to determine not only participation but also the local learning configuration, including training intensity and lightweight learning operations.

### 2.2. Energy-Aware Federated Learning

Recent work has explored energy-aware federated learning to improve learning efficiency under resource-constrained environments [4,8,9]. A widely adopted approach is client selection, where only devices with sufficient resources are chosen to participate in each training round [10]. This technique reduces the risk of training interruption and improves convergence stability.

In addition, adaptive participation strategies have been proposed, allowing devices to dynamically decide whether to participate based on their current energy conditions [11]. These approaches improve system robustness by preventing energy-deficient devices from participating in costly training processes. However, most of these methods primarily focus on participation control. Once a client is selected, the learning process itself is typically performed using a fixed configuration. As a result, these approaches do not fully utilize the available energy resources, especially in environments where energy availability fluctuates significantly over time.

More recent studies have considered energy-harvesting-aware federated learning under dynamic energy supply and wireless communication conditions. Zhang et al. formulated federated learning with energy-harvesting devices as a Markov decision process and analyzed the effect of partial device participation and packet drops on convergence [12]. Their framework jointly optimizes device scheduling and transmission power by considering battery and channel states. Furthermore, they developed a structure-enhanced deep-reinforcement-learning algorithm for unknown channel and energy-harvesting statistics. This study is highly relevant to EH-FL because it explicitly models the dependence of participation and packet delivery on the available energy supply. However, its main control variables are device scheduling and transmission power. In contrast, the proposed framework focuses on the learning-configuration level: after a client is considered eligible for participation, the framework determines how local training should be performed by jointly controlling local epochs, batch size, model pruning, quantization, and knowledge distillation under the same available energy constraint.

Battery-aware scheduling has also been investigated in regard to energy-harvesting federated learning. Jeong and Pappas proposed a battery-aware cyclic scheduling framework in which client participation is coordinated according to battery availability in EH-FL systems [23]. Such scheduling-oriented approaches are effective for improving participation continuity and reducing energy-related training failures. Nevertheless, their primary objective is to determine when or which clients should participate in federated learning. They do not explicitly adapt the internal learning configuration of each participating client. By contrast, the proposed method uses the residual and harvested energy state of each client to determine not only participation but also the feasible local learning configuration, thereby extending energy awareness from client-level scheduling to computation-level learning control.

Energy-efficient edge-learning studies have also explored adaptive training activation according to devices’ operating states. For example, Asad and Otoum proposed an adaptive idle-time training approach that considers battery state, charging state, and resource availability when activating federated learning on edge nodes [24]. This approach is related to energy-aware FL because it reduces unnecessary local training under unfavorable device conditions. However, its main focus is training activation, whereas the proposed framework further adapts the internal learning configuration of participating clients under the available energy budget.

Overall, existing energy-aware and energy-harvesting-aware FL studies have mainly formulated energy adaptation as a problem of client selection, participation timing, device scheduling, transmission power control, or training activation. These directions are important for sustainable FL, but they do not fully address how the internal learning process of each participating client should be configured under time-varying harvested energy. The proposed framework differs from these studies by formulating EH-AIoT federated learning as a unified learning-configuration control problem, where participation, training intensity, model complexity, and computational precision are jointly coordinated according to the available learning energy of each client.

### 2.3. Model Compression and Efficient Learning Techniques

Various model compression techniques have been devised to reduce the computational and communication overhead of federated learning. Model pruning removes redundant parameters from deep neural networks, thereby reducing model size and computational cost [13,14]. Quantization reduces numerical precision to improve computational efficiency and memory usage [15,16].

In addition, approximate computing techniques have been explored to further improve energy efficiency in edge AI systems [17,18,19]. These techniques allow systems to trade computational accuracy for reduced energy consumption, making them suitable for resource-constrained environments.

Knowledge distillation has also been applied to federated learning to transfer knowledge from large global models to lightweight local models [20]. This approach enables resource-constrained devices to maintain competitive performance despite reduced model complexity.

Despite their effectiveness, these efficient learning techniques are typically applied independently. Researchers usually apply pruning, quantization, approximate computing, or knowledge distillation for model efficiency improvement, but they do not sufficiently consider how these techniques should be coordinated with adaptive training intensity under dynamically changing energy conditions. In contrast, the proposed framework explicitly couples lightweight learning operations with adaptive training intensity control under the same energy-aware configuration selection process.

### 2.4. Research Gap and Distinction of the Proposed Framework

Although significant progress has been made in energy-aware federated learning, energy-harvesting-aware FL, and efficient model optimization, existing approaches still leave an important research gap for EH-AIoT systems. Most energy-aware FL studies primarily focus on client selection, participation timing, resource scheduling, or communication control. These approaches determine whether a client should participate, when it should participate, or how communication resources should be allocated. However, they generally do not address how the internal learning configuration of each participating client should be adapted under dynamically changing harvested energy.

As summarized in Table 1, existing studies can be broadly categorized into scheduling-oriented, communication-oriented, activation-oriented, and model-efficiency-oriented approaches. Energy-aware FL studies have mainly addressed client selection and resource allocation, while energy-harvesting-aware FL studies have further incorporated battery states, harvested energy availability, wireless channel conditions, packet drops, and transmission power control. These studies are important because they explicitly recognize that energy availability affects the feasibility and reliability of federated learning in practical systems. Nevertheless, their main control targets remain participation, scheduling, communication, or training activation.

In contrast, the proposed framework focuses on the learning-configuration level. Once a client becomes eligible for participation, the proposed method determines how the local learning process should be performed under the available learning energy. Specifically, the available learning energy is used as a common constraint for jointly selecting local training intensity, such as epoch size and batch size, and lightweight learning operations, such as model pruning, quantization, and knowledge distillation. Therefore, the proposed method does not simply add model compression to an energy-aware FL baseline; rather, it organizes participation control, computation control, model-efficiency control, and performance-recovery control within a single energy-adaptive decision structure.

This limitation also applies to efficient learning techniques such as pruning, quantization, approximate computing, and knowledge distillation. However, these techniques are often introduced as separate model-compression or efficiency mechanisms. They are not usually coordinated with energy-aware participation control and adaptive local training intensity under the same available-energy constraint. Therefore, existing studies do not fully capture the interaction among participation, computation amount, model complexity, numerical precision, and performance recovery, although these factors jointly determine both energy consumption and learning effectiveness in EH-AIoT environments.

The proposed framework addresses this gap by formulating energy-aware federated learning as a unified multi-dimensional learning-configuration control problem. The key distinction is that the available learning energy of each client is used not only to determine whether the client participates, but also to determine how local learning is performed. Specifically, the proposed method jointly coordinates participation, local training intensity, model pruning, quantization, and knowledge distillation within a single energy-adaptive decision structure. This extends energy awareness from binary participation or scheduling control to configuration-level learning control.

Therefore, the novelty of the proposed framework does not lie in the individual use of an energy-threshold rule, pruning, quantization, or knowledge distillation. Rather, its main contribution is the unified organization of these components under a common available-energy constraint. This distinction directly connects the related work discussion with the research gap addressed in this paper and supports the claimed contribution of multi-dimensional learning control for federated learning in EH-AIoT systems.

## 3. Proposed Method

This section presents the proposed energy-adaptive multi-dimensional learning control framework in detail. The main objective of the proposed method is to enable stable and efficient federated learning in energy-harvesting AIoT environments, where the energy available to each device varies continuously over time. In such environments, a device cannot always participate in local training with a fixed learning configuration because the same amount of computation may be affordable in one round but cause blackout in another. Therefore, the learning procedure itself must be adapted to the energy state of each device.

The proposed framework is designed based on the following principle: each client should use as much energy as possible for learning when sufficient harvested energy is available but also avoid excessive consumption that may destabilize future operation. To realize this goal, the framework makes three coordinated decisions in every federated learning round. First, each client decides whether it can safely participate in local training. Second, if the client participates, it determines the intensity of local training by adjusting the number of epochs and batch size. Third, the client applies lightweight learning techniques, including model pruning, quantization, and knowledge distillation, to reduce the learning cost while maintaining model quality. These decisions are not made independently; rather, they are jointly determined according to the client’s real-time energy state. 

**Figure 2:**
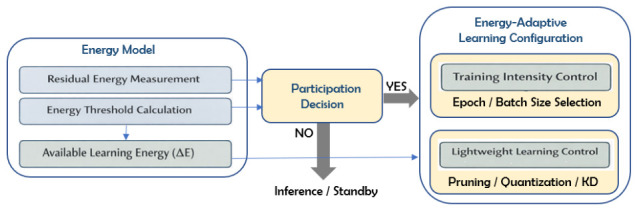
Overview of the proposed energy-adaptive multi-dimensional learning control framework. Each client first determines its participation based on the available learning energy (ΔE). If participation is allowed, the learning configuration is adaptively determined by jointly considering training intensity and lightweight learning operations.

A decision flowchart for the proposed framework is illustrated in Figure 2. As shown in the figure, each client first evaluates its energy condition through the energy model, which computes the available learning energy (ΔE). Based on this value, the client determines whether to participate in the current federated learning round. If participation is not allowed, the device switches to inference or standby mode to preserve energy. Otherwise, the client adaptively determines its learning configuration by jointly considering training intensity (e.g., epoch size and batch size) and lightweight learning operations, including pruning, quantization, and knowledge distillation. This structured decision flow ensures that the learning process remains both energy-efficient and stable under dynamically changing energy conditions.

### 3.1. System Overview

As illustrated in Figure 1, the proposed framework consists of one central server and multiple energy-harvesting AIoT clients. The central server maintains the global model and aggregates model updates received from participating clients. Each client is equipped with an energy-harvesting power source, such as a solar panel, a rechargeable battery, and an energy-aware controller. The energy-aware controller continuously monitors the device-level energy status and determines how much learning can be performed in the current round.

At the beginning of each federated learning round, the server distributes the current global model to the clients. Then, each client measures its residual energy and estimates whether it can safely conduct local training during the round. If the client determines that local training is unsafe under the current energy conditions, it skips training and only continues inference or standby operation. If the energy condition is sufficient, the client participates in federated learning and chooses an energy-adaptive learning configuration. In the proposed framework, this configuration includes the local training intensity (epoch size and batch size) and lightweight learning operations (pruning, quantization, and knowledge distillation). After local training, the client sends its model update to the server, and the server aggregates the received updates to construct the next global model.

This design differs from conventional energy-aware client selection methods. In conventional methods, energy is mainly used to decide whether a client participates. In the proposed framework, however, energy is used not only for participation control but also for determining how training itself should be performed. This enables finer-grained adaptation and makes the overall framework more robust to energy variability.

### 3.2. Energy Model and Participation Decision

The first step of the proposed method is to determine whether a client can safely participate in local training in the current round. For this purpose, we adopt an energy-threshold-based decision model. The purpose of this model is not to predict the exact amount of future harvested energy over a long horizon but to identify whether the current device state allows local training without significantly increasing the risk of a blackout.

Let $E_{i}^{\left(t\right)}$ denote the residual energy stored in the battery of client *i* in round *t*, and let $C_{i}$ denote the total battery capacity of client *i*. We further define $P_{solar,i}^{\left(t\right)}$ as the average harvested power and $P_{sys,i}^{\left(t\right)}$ as the average system power consumption of client *i* measured over the current operating window. These values are obtained from device-level energy monitoring during the recent operating period and used as lightweight online estimates for energy-aware decision making under the current operating condition.

Following the energy-threshold formulation introduced in our previous energy-aware EH-IoT design study [21], the time required to fully charge the battery can be expressed as follows:

(1) $$T_{\mathrm{full},i}^{\left(t\right)}\left(E_{i}^{\left(t\right)}\right)=\frac{C_{i}-E_{i}^{\left(t\right)}}{P_{solar,i}^{\left(t\right)}-P_{sys,i}^{\left(t\right)}}.$$

The formulation above provides an intuitive interpretation of the charging behavior under the current operating condition. Specifically, the charging time depends on the balance between harvested power and system power consumption. If the amount of power harvested is sufficiently greater than system power consumption, the battery can recover more quickly, allowing additional energy to be allocated to local learning.

Based on this observation, the energy threshold of client *i* in round *t* can be expressed as follows:

(2) $$E_{\mathrm{th},i}^{\left(t\right)}=\frac{P_{sys,i}^{\left(t\right)}}{P_{solar,i}^{\left(t\right)}}C_{i}.$$

This threshold can be interpreted as an energy-aware safety boundary for local learning. If the residual energy is greater than the threshold, the client is considered to be in a relatively safe operating region where additional energy can be allocated to local training without significantly increasing blackout risk. In contrast, if the residual energy falls below the threshold, the client enters an energy-deficient region where aggressive computation may destabilize future operation.

Based on this threshold, we define the available learning energy margin of client *i* in round *t* as follows:

(3) $$\Delta E_{i}^{\left(t\right)}=E_{i}^{\left(t\right)}-E_{\mathrm{th},i}^{\left(t\right)}.$$

The proposed participation policy is intentionally simple and interpretable. If $\Delta E_{i}^{\left(t\right)}\leq 0$, client *i* does not participate in local training during round *t*. Instead, it preserves energy and continues only lightweight operation, such as inference or idle standby. If $\Delta E_{i}^{\left(t\right)}>0$, client *i* becomes eligible for local training. In other words, the proposed method does not force every device to train in every round. Rather, it allocates training only to the clients who can afford it under their current energy condition.

This participation decision is important because blackout avoidance is not merely an implementation convenience; it is directly related to the long-term stability of the learning system. If a client repeatedly trains under unsafe energy conditions, it may be unavailable in subsequent rounds, reducing participation continuity and degrading the quality of the global model. Therefore, the threshold-based decision serves as the first safety barrier of the entire framework.

It is important to note that the proposed threshold is designed as a lightweight online decision criterion based on recently measured operating conditions rather than a long-term predictive energy model. Therefore, the threshold dynamically changes according to the current harvested power and system power consumption conditions, enabling adaptive behavior under varying environmental conditions such as solar intensity variation and changing workload patterns.

While the proposed threshold model provides an interpretable and computationally efficient decision mechanism, more sophisticated prediction-based energy management approaches may further improve long-term optimization in highly dynamic energy environments. Exploring such predictive strategies remains an important direction for future work.

### 3.3. Energy-Adaptive Training Intensity Control

Once a client decides to participate in local training, the next question is: How much training should be performed? Even among participating clients, the available energy margin $\Delta E_{i}^{\left(t\right)}$ can differ significantly. A client with a large margin can contribute more aggressively to learning, whereas a client with a small margin should perform only lightweight updates. To capture this behavior, the proposed method controls the local training intensity through two parameters: the number of local epochs and batch size.

The motivation is straightforward. The number of local epochs determines how many times the local dataset will be revisited, and batch size affects both the computational pattern and per-round energy consumption. Increasing either parameter generally improves the amount of local learning but also increases the energy cost. Therefore, the training intensity should be matched to the energy budget of the client.

Formally, let $K_{i}^{\left(t\right)}$ and $B_{i}^{\left(t\right)}$ denote the selected number of local epochs and batch size of client *i* in round *t*, respectively. The proposed method assumes that each client maintains a lightweight profiling table obtained offline in advance. This table records the approximate energy consumption associated with candidate training configurations such as $\left(K,B\right)\in C_{train}$, where $C_{train}$ is a finite set of feasible epoch–batch pairs. In round *t*, the client searches this table and selects the strongest configuration whose expected cost does not exceed the available learning energy margin:

(4) $$\left(K_{i}^{\left(t\right)},B_{i}^{\left(t\right)}\right)=\arg\max_{\left(K,B\right)\in C_{train}}U\left(K,B\right)\;s.t.\;{\hat{E}}_{train}\left(K,B\right)\leq \Delta E_{i}^{\left(t\right)},$$

where ${\hat{E}}_{train}\left(K,B\right)$ is the profiled training energy cost and U(K,B) is a utility score reflecting the expected learning benefit of the configuration.

In the proposed framework, the utility function is designed to favor learning configurations that provide stronger local learning performance while remaining feasible under the available energy constraint. In practice, the utility score may increase with larger local epochs and more effective batch sizes, since these generally improve local model updates at the cost of higher energy consumption.

In each federated learning round, the client searches the candidate configuration set $C_{train}$ and evaluates all feasible configurations satisfying

$${\hat{E}}_{train}\left(K,B\right)\leq \Delta E_{i}^{\left(t\right)}.$$

Among these feasible candidates, the client selects the configuration with the highest utility score. Therefore, the proposed decision mechanism can be interpreted as a constrained utility-maximization process under the available learning energy margin.

This trade-off is important because aggressive local training may improve learning performance but can also increase the risk of future blackout. Conversely, overly conservative configurations may preserve energy but also contribute insufficiently to global learning. The proposed framework balances these competing objectives by adaptively selecting training intensity according to the current energy condition of each client.

The exact form of U(K,B) may vary depending on the implementation. In the simplest form, the client may prefer larger epochs first and then choose the largest feasible batch size. The important point is that the selected configuration must remain feasible under the current energy condition. This mechanism ensures that the client uses available energy productively without crossing the safety boundary defined by the threshold model.

This design is more suitable than a fixed training configuration because it naturally adapts to dynamic energy conditions. When a great amount of energy has been harvested, the client can choose larger epochs and a more effective batch size, thereby increasing its contribution to global learning. When energy is scarce, the client still participates, but with reduced training intensity. In addition, the proposed framework jointly considers lightweight learning operations together with training-intensity control. For example, clients with limited available learning energy may employ smaller epoch and batch-size configurations together with stronger pruning and more aggressive quantization in order to reduce computation and communication costs. In contrast, clients with sufficient energy margins can employ greater training intensity with less aggressive model compression to improve learning effectiveness. This adaptive configuration behavior enables more flexible trade-offs between learning effectiveness and energy efficiency under dynamically changing energy conditions. As a result, the global training process becomes smoother and less sensitive to temporary energy shortages.

However, it is important to note that the proposed framework does not treat training-intensity control and lightweight learning as independent sequential steps. Instead, they are conceptually coupled and jointly determined under the same energy constraint. In other words, each client selects a feasible learning configuration that satisfies the available learning energy ΔE, where both training-intensity parameters (e.g., epoch size and batch size) and model efficiency techniques (e.g., pruning, quantization, and knowledge distillation) are considered together.

From this perspective, the selection of $\left(K_{i}^{\left(t\right)},B_{i}^{\left(t\right)}\right)$ in Equation (4) can be interpreted as being part of a broader configuration decision rather than being an isolated optimization of training iterations. This joint consideration enables the client to balance computation cost and model complexity more effectively, allowing a wider range of feasible solutions under dynamic energy conditions.

### 3.4. Lightweight Learning via Model Pruning, Quantization, and Knowledge Distillation

Specifically, given the available learning energy ΔE, each client selects a feasible learning configuration that satisfies the energy budget by jointly considering both the amount of computation and the cost per computation. This joint design allows the client to balance learning effectiveness and energy efficiency more flexibly, enabling a wider range of feasible solutions relative to approaches that adjust only a single dimension.

To further improve energy efficiency at the model level, the proposed framework employs three lightweight learning techniques: model pruning, quantization, and knowledge distillation.

#### 3.4.1. Model Pruning

Model pruning is used to eliminate unnecessary parameters in the local model to reduce both training and inference costs. The role of pruning in the proposed framework is not merely offline compression; it is used as an adaptive cost-control mechanism for energy-constrained clients.

Let $M_{full}^{\left(t\right)}$ denote the original global model distributed by the server in round *t*. For each participating client, a pruned local model $M_{pruned,i}^{\left(t\right)}$ is generated by removing parameters or channels with low importance. In practice, the pruning operation may follow a structured pruning rule such as network slimming or channel pruning so that the resulting model remains hardware-friendly. The pruning level can be predetermined or selected from a small set of candidate levels according to the client energy condition.

The logic is intuitive. A heavily pruned model has lower computational costs and a smaller update size, which are desirable features when energy is limited. On the other hand, an overly aggressive pruning ratio may reduce model capacity and damage accuracy. Therefore, pruning serves as a controllable trade-off parameter between energy efficiency and learning performance. In the proposed framework, pruning is combined with threshold-based energy control so that clients with less available learning energy are more likely to use lighter local models. 

#### 3.4.2. Quantization

Quantization is used to reduce the numerical precision of model parameters and intermediate computations. Its main benefit is that it reduces memory usage, arithmetic cost, and communication size. In the proposed implementation, low-precision representation such as INT8 is employed for local learning and update transmission whenever possible.

From the perspective of energy-aware learning, quantization complements pruning. While pruning reduces the structural complexity of the model, quantization reduces the representation cost of each remaining parameter. As a result, the combined effect can substantially reduce the end-to-end cost of local training. In addition, since federated learning requires repeated communication between the server and the clients, shrinking model updates through quantization directly helps to lower transmission energy.

In the proposed framework, quantization is treated as a standard lightweight learning option for participating clients. Once a client is selected for training, it performs local optimization using the quantized model representation and transmits compressed updates to the server. This process makes the learning pipeline more feasible for EH-powered devices without requiring substantial changes to the global federated learning protocol.

#### 3.4.3. Knowledge Distillation

Pruning and quantization improve efficiency, but they may also degrade the expressive power of the local model. To compensate for this loss, the proposed method incorporates knowledge distillation. The basic idea is to transfer knowledge from a stronger teacher model to a lightweight student model so that the student can preserve as much predictive capacity as possible despite its reduced complexity.

In the proposed framework, the server-side global model acts as the teacher, and the client-side lightweight model acts as the student. Let $z_{T}\left(x\right)$ and $z_{S}\left(x\right)$ denote the teacher and student logits for input sample *x*, respectively. The student model is trained not only with the conventional task loss but also with a distillation loss that encourages it to mimic the teacher’s behavior. A common form of the distillation loss is

(5) $$L_{KD}=D_{KL}\left(\sigma \;\left(\frac{z_{T}\left(x\right)}{\tau }\right)\;∥\;\sigma \;\left(\frac{z_{S}\left(x\right)}{\tau }\right)\right),$$

where σ(·) denotes the softmax function, τ is the temperature parameter, and $D_{KL}$ is the Kullback–Leibler divergence.

The final local objective of client *i* in round *t* can be written as

(6) $$L_{i}^{\left(t\right)}=L_{task,i}^{\left(t\right)}+\lambda_{KD}L_{KD,i}^{\left(t\right)},$$

where $L_{task,i}^{\left(t\right)}$ is the standard task-specific loss and $\lambda_{KD}$ is a balancing coefficient.

The role of knowledge distillation in the proposed method is important. Instead of treating model compression as a pure efficiency operation, the framework uses distillation to recover part of the performance that may be lost due to pruning and quantization. This approach allows each client to remain lightweight while still contributing useful updates to the global model.

### 3.5. Unified Multi-Dimensional Learning Control

Notably, the novelty of the proposed framework is not due to any individual component in isolation. The energy-threshold model, pruning, quantization, and knowledge distillation are known techniques or design principles. In the proposed framework, however, they are organized into a unified energy-adaptive configuration-control structure, where the role of energy awareness is extended from binary participation control to unified multi-dimensional learning-configuration control.

In conventional energy-aware federated learning approaches, energy is typically used only to determine whether a client participates in training. In contrast, the proposed framework extends the role of energy awareness to the configuration level. Specifically, the framework jointly determines not only client participation but also training intensity and lightweight learning operations according to the available learning energy of each client.

In each round, a client first computes its available learning energy and determines whether local training is safe. If participation is allowed, the client then selects a feasible learning configuration under the same energy constraint. This configuration jointly specifies both training intensity parameters (e.g., epoch size and batch size) and lightweight learning operations (e.g., pruning, quantization, and knowledge distillation). As a result, the proposed method transforms energy awareness from a binary participation rule into a unified multi-dimensional learning-configuration control mechanism.

This distinction is important because the proposed framework does not simply combine existing lightweight learning techniques in a sequential manner. Instead, it explicitly couples computation amount, model complexity, and computational precision within a single energy-adaptive decision process. This unified control perspective enables more flexible adaptation to dynamic energy conditions and represents the main novelty of the proposed framework compared with existing energy-aware FL approaches.

Another important aspect of the proposed framework is that the decision process is explicitly structured as a constrained configuration selection procedure. Rather than independently applying pruning, quantization, or training adaptation after participation is determined, the proposed framework jointly evaluates these learning dimensions under the same available learning energy constraint. This explicit decision structure improves reproducibility and provides clearer configuration selection logic for energy-aware federated learning in EH-AIoT environments.

### 3.6. Federated Learning Procedure

The overall steps of the proposed framework are summarized in Algorithm 1. In every federated learning round, the server broadcasts the current global model. Each client computes its energy margin and decides whether to participate. Participating clients then determine a feasible learning configuration under the given energy constraint. This configuration jointly specifies both the training intensity (e.g., epoch size and batch size) and the lightweight learning operations (e.g., pruning, quantization, and knowledge distillation). Based on this configuration, each client performs local training and transmits the resulting model update. The server aggregates the received updates using a standard federated aggregation rule such as FedAvg.

### 3.7. Design Rationale and Implementation Considerations

The proposed method is designed to be understandable, implementable, and robust. It is understandable because each control stage has a clear physical role: thresholding prevents unsafe training, while the learning configuration is determined under the available energy budget by jointly considering training intensity and lightweight learning operations. This unified view allows the framework to transcend mere sequential control and instead adapt the entire learning process to the current energy condition.

It is implementable because the framework relies on device-level measurements and a small profiling table rather than a complex long-term energy prediction model. It is also robust because it can adapt to continuously changing energy conditions without requiring all clients to behave identically.

Another important point is that the proposed framework does not assume that all clients must use the same learning configuration. On the contrary, heterogeneity is a fundamental design assumption. Different clients may have different amounts of residual energy and harvested power and different feasible training budgets in the same round. The proposed method explicitly embraces this heterogeneity and converts it into an adaptive learning strategy.

For these reasons, the proposed energy-adaptive multi-dimensional learning control framework is well-suited to federated learning in energy-harvesting AIoT systems, where both learning performance and operational stability must be considered simultaneously.
**Algorithm 1:** Energy-Adaptive Multi-Dimensional Learning Control for Federated Learning.
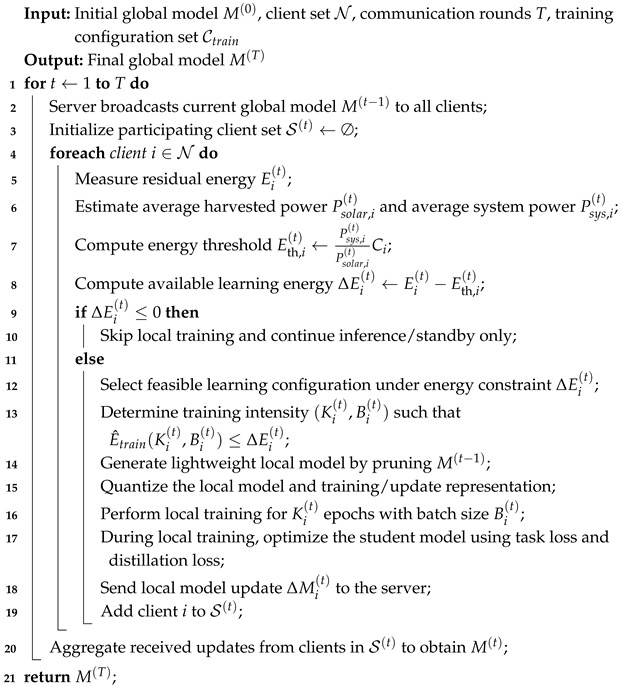


## 4. Experimental Results

This section evaluates the proposed energy-adaptive multi-dimensional learning control framework in a realistic energy-harvesting AIoT environment. The experiments were designed to validate the framework from multiple perspectives, including overall learning performance, blackout reduction, participation continuity, component-level contribution, and temporal stability under time-varying energy availability.

Although the testbed was intentionally kept at a manageable scale to enable controlled measurement on real edge devices, our evaluation covers several complementary aspects of the proposed framework. We first describe the experimental setup and compare methods and then present an overall performance comparison, an ablation analysis, and a temporal behavior analysis to demonstrate the practical usefulness of the proposed method.

### 4.1. Setup of the Experiment

To validate the proposed framework, we constructed a real-world testbed consisting of one federated learning server and five energy-harvesting AIoT clients. Table 2 summarizes the main experimental parameters, including the testbed configuration, dataset settings, training parameters, and model efficiency options used in the evaluation. Each client was implemented on an NVIDIA Jetson Orin Nano device powered by a solar panel and a rechargeable battery. The clients communicated with the server through Wi-Fi, and the power status of each device was monitored using jetson_stats.

The CIFAR-10 dataset was used for image classification under a non-IID setting, where each client possessed data from two classes. The global model was based on ResNet-18, and each client used a lightweight student model obtained via pruning and quantization. Knowledge distillation was applied during local training.

Each federated learning round corresponded to one day, and the experiment was conducted for 15 days. The harvested energy varied between 150 Wh and 400 Wh depending on environmental conditions. The battery capacity of each client was fixed at 240 Wh.

The reported learning energy refers only to the energy consumed for local training and model updating, excluding baseline system power consumption.

### 4.2. Methods Compared

To evaluate the effectiveness of the proposed framework, we compared it with several baseline methods that represent different levels of energy awareness and model optimization in federated learning.

- Ideal: All clients always participate with sufficient energy, serving as an upper-bound reference without practical energy constraints.
- All_Nodes: All clients participate regardless of energy conditions, representing a naive baseline without energy-aware participation control.
- Threshold-Only: Clients participate only when ΔE>0 but use a fixed training configuration. This baseline represents a lightweight energy-aware participation-control approach in which participation is adaptively determined according to the available learning energy.
- Pruning-Only: This approach extends Threshold-Only by additionally applying lightweight model compression through pruning without jointly adapting training intensity or other learning configurations.
- Proposed: This is the full framework, which jointly coordinates participation, training intensity, and lightweight learning operations under the same energy constraint.

### 4.3. Overall Performance Comparison

Table 3 presents a comparison of the overall performance of the proposed method and baseline approaches. The Ideal case achieved the highest accuracy, 91.5%, as all clients continuously participated in training without any energy constraints. However, this scenario is not feasible in practical energy-harvesting environments.

The All_Nodes method showed the worst performance, achieving only 73.4% accuracy with a very high blackout ratio of 58.7%. Although all clients initially participated, the lack of energy-aware control led to excessive energy consumption, causing frequent blackouts and unstable training. The Threshold-Only method reduced blackout to 10.4% and increased participation to 57.6%. However, its fixed training configuration limited learning efficiency, resulting in only moderate accuracy. The Pruning-Only method further improved stability, reducing blackout to 4.3% and increasing accuracy to 84.6%. This confirms that model compression effectively reduces training cost, but the lack of adaptive control limits performance.

The proposed method achieved the best trade-off, reaching 87.2% accuracy while maintaining a low blackout ratio of 3.1%. These results demonstrate that joint control of training intensity and lightweight learning enables more effective energy utilization. Overall, the results suggest that simply reducing model complexity or limiting participation is insufficient and that coordinated control across multiple dimensions is necessary to fully exploit the available energy.

From a validation perspective, these results show that the proposed framework improves multiple performance aspects simultaneously rather than optimizing only a single metric. In particular, the proposed method achieves higher accuracy than the energy-aware baseline methods while maintaining substantially lower blackout under energy-harvesting constraints. This finding confirms that the proposed joint control mechanism is effective not only for reducing energy depletion but also for preserving useful learning participation under dynamic energy conditions.

### 4.4. Ablation Study

Table 4 summarizes the ablation study. The results show that each component contributes incrementally. Adaptive training improves energy utilization, while pruning and quantization reduce computational cost. Knowledge distillation further improves accuracy by compensating for compression loss.

Furthermore, the results highlight the importance of joint control. While each component individually improves performance, their combination provides additional gains beyond the sum of the individual effects. This indicates that the interaction between training intensity and lightweight learning plays a critical role in energy-efficient federated learning under dynamic energy conditions.

These ablation results further validate the proposed framework by showing that the performance gain is not a product of a single component. Instead, the improvements are obtained through the coordinated interaction among adaptive intensity control, model compression, and knowledge distillation. This finding supports the main design principle of the proposed framework, namely, that energy-aware federated learning should be treated as a multi-dimensional control problem.

### 4.5. Temporal Behavior

**Figure 3:**
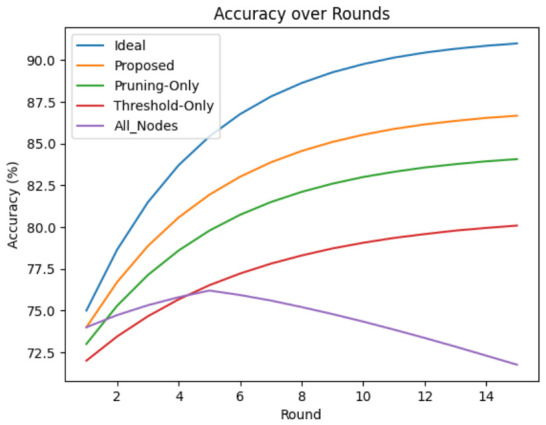
Accuracy evolution over time. The proposed method shows stable convergence close to the Ideal case.

Figure 3 illustrates the evolution of accuracy over time. The Ideal case converges to the highest accuracy. The All_Nodes method becomes unstable due to frequent blackouts. The Threshold-Only method converges slowly, while the proposed method achieves stable and efficient convergence. This behavior indicates that the proposed method effectively mitigates the impact of energy fluctuations on learning stability.

**Figure 4:**
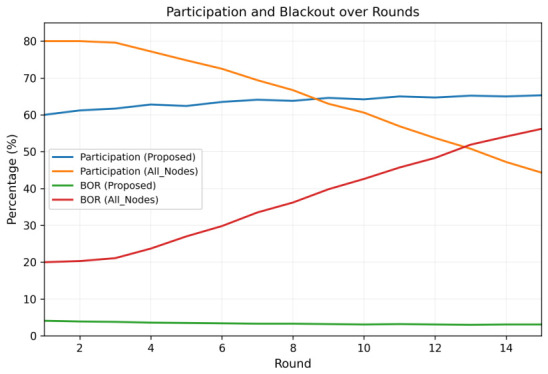
Participation and blackout behavior over time. The proposed method maintains stable participation and low blackout. For clarity, only the proposed and All_Nodes methods are shown.

Figure 4 shows participation and blackout trends. The proposed method maintains stable participation while keeping blackout low, unlike All_Nodes, which shows severe instability due to excessive energy consumption. This result indicates that the proposed joint control mechanism effectively balances energy usage across clients, preventing abrupt energy depletion and enabling more consistent participation over time.

As a result, the proposed framework improves not only system stability but also the overall effectiveness of federated learning under dynamic energy conditions.

### 4.6. Discussion of Experimental Results

The results confirm three key notions. First, participation control alone is insufficient because although it can reduce blackout, it cannot fully utilize the available learning opportunity. Second, lightweight learning reduces computational cost, but its effectiveness is limited when it is not coordinated with adaptive training intensity. Third, knowledge distillation is useful for preserving model accuracy when pruning and quantization reduce model capacity.

These observations indicate that the proposed framework provides a broader validation than a single performance comparison. The overall comparison demonstrates the trade-off between accuracy, blackout, participation, and learning energy. The ablation study verifies the contribution of each control component, while the temporal analysis shows that the proposed method maintains more stable behavior over time under dynamic energy conditions.

Nevertheless, the current validation has limitations. The experiments were conducted on a small-scale testbed with five Jetson Orin Nano clients, which we selected to enable controlled real-device measurement under energy-harvesting conditions. Larger-scale deployments with more heterogeneous devices, diverse energy-harvesting profiles, and longer evaluation periods may further demonstrate the general applicability of the proposed framework.

In addition, the proposed framework is structurally suitable for heterogeneous EH-AIoT environments because each client independently determines its feasible learning configuration according to local energy conditions and resource availability. Since the framework relies on lightweight online configuration decisions rather than centralized global optimization or scheduling, it can naturally adapt to dynamically changing client conditions without requiring homogeneous client behavior.

The Threshold-Only method can also be interpreted as a lightweight energy-aware participation-control baseline because client participation is adaptively determined according to the available learning energy. In contrast to scheduling-oriented approaches that primarily focus on participation control or resource allocation, the proposed framework focuses on jointly coordinating training intensity and lightweight learning operations under the same energy constraint. More extensive comparisons with larger-scale scheduling-oriented energy-aware FL methods and advanced adaptive client-selection approaches remain important directions for future work.

Overall, the proposed framework demonstrates that energy-aware federated learning should be treated as a multi-dimensional control problem rather than a single participation-control problem. Our experimental results provide practical evidence that joint control of participation, training intensity, and lightweight learning operations can improve learning stability and energy efficiency in EH-AIoT environments.

## 5. Conclusions

In this paper, we propose an energy-adaptive multi-dimensional learning control framework for federated learning in energy-harvesting AIoT systems. Unlike conventional approaches that consider only a single aspect of learning cost, the proposed method jointly controls multiple dimensions, including client participation, training intensity, and lightweight learning operations under a unified energy constraint.

The proposed framework enables each client to determine its learning behavior based on its real-time energy condition. By incorporating an energy-threshold-based participation decision, adaptive training intensity control, and lightweight learning techniques such as model pruning, quantization, and knowledge distillation, the framework effectively balances energy consumption and learning performance. In particular, the joint consideration of training intensity and model efficiency allows each client to select a feasible learning configuration that maximizes its contribution while preventing energy depletion.

The results of our experiments conducted on a real-world AIoT testbed demonstrate that the proposed method can achieve stable and efficient learning under time-varying energy conditions. Compared to baseline methods, the proposed framework significantly reduces device blackout while maintaining competitive model accuracy. The ablation study further confirmed that each component contributes to performance improvement and that only their joint integration leads to the best overall results.

The main contribution of this work lies in the formulation of energy-aware federated learning in EH-AIoT systems as a unified multi-dimensional configuration control problem rather than a simple participation decision problem. Unlike conventional approaches that separately optimize model compression, training intensity, or participation, the proposed framework jointly coordinates these learning dimensions under a common energy constraint.

By explicitly coupling computation amount, model complexity, and computational precision according to the real-time energy state of each client, the proposed framework enables more flexible adaptation to dynamic energy conditions. The experimental results demonstrate that this unified control perspective not only improves system stability but also maintains competitive learning performance under energy-constrained environments.

For future work, more advanced learning configuration strategies can be explored, such as incorporating predictive energy models or optimizing the configuration selection process using reinforcement learning. In addition, extending the proposed framework to larger-scale deployments with more heterogeneous devices remains an important direction for further research.

## Figures and Tables

**Table 1:** Comparison of existing energy-aware and energy-harvesting-aware FL studies with the proposed framework.

Research Direction	Main Control Target	Limitation with Respect to EH-AIoT Learning Control	Difference of This Work
Energy-aware FL and resource management [8,10,11]	Client selection, resource allocation, device-to-device assistance, or heterogeneous-resource-aware participation	Energy awareness is mainly used to determine client participation or resource allocation, while the local learning configuration is often fixed after selection.	The proposed method extends energy awareness from participation control to local learning-configuration control.
Energy-harvesting-aware FL [12,23]	Battery-aware scheduling, harvested-energy-aware participation, channel-aware transmission, packet-drop handling, or transmission power control	The main focus is scheduling or communication control under EH dynamics; the internal learning process of participating clients is not jointly configured.	The proposed method uses available learning energy to jointly determine participation and local learning behavior.
Energy-efficient training activation [24]	Activation of local training according to battery state, charging state, idle time, or device resource availability	The method determines whether local training should be activated, but does not fully coordinate training intensity and model-efficiency operations.	The proposed method further controls how local training is performed after participation is allowed.
Model compression and efficient learning [13,14,15,16,20]	Pruning, quantization, or knowledge distillation for reducing computation, memory, or communication cost	These techniques are usually applied as separate efficiency mechanisms and are not jointly coordinated with EH-aware participation and adaptive training intensity.	The proposed method treats pruning, quantization, and knowledge distillation as controllable learning dimensions under the same energy constraint.
Proposed framework	Participation, local epochs, batch size, pruning, quantization, and knowledge distillation	–	Unified multi-dimensional learning-configuration control under available learning energy.

**Table 2:** Main experimental parameters.

Parameter	Value
FL server	DELL PowerEdge R760XA (Round Rock, TX, USA)
Client device	NVIDIA Jetson Orin Nano (5 clients) (Santa Clara, CA, USA)
Battery capacity	240 Wh
Harvested energy	150–400 Wh/day
Global model (teacher)	ResNet-18
Local model (student)	Pruned and quantized ResNet-18
Dataset	CIFAR-10 (non-IID, 2 classes per client)
Round duration	1 day
Experiment duration	15 days
Federated aggregation	FedAvg
**Training Parameters**	
Optimizer	SGD
Learning rate	0.001
Momentum	0.9
Candidate epochs *K*	{1, 2, 3, 4}
Candidate batch sizes *B*	{16, 32, 64}
**Model Efficiency**	
Pruning ratio	30% (structured pruning)
Quantization	INT8
**Knowledge Distillation**	
Distillation loss	KL divergence
Temperature τ	4
Weight $\lambda_{KD}$	0.5

**Table 3:** Comparison of the performance of different methods.

Method	Accuracy (%)	BOR (%)	Participation (%)	Learning Energy (J)
Ideal	**91.5**	**0.0**	**100.0**	341
All_Nodes	73.4	58.7	39.2	356
Threshold-Only	80.8	10.4	57.6	301
Pruning-Only	84.6	4.3	60.3	238
Proposed	**87.2**	**3.1**	**64.8**	**247**

**Table 4:** Ablation study of the proposed framework.

Variant	Adaptive Intensity	Pruning/Quant.	KD	Accuracy (%)	BOR (%)
Participation-Only	×	×	×	80.8	10.4
+Adaptive Intensity	✓	×	×	83.1	7.6
+Pruning + Quantization	✓	✓	×	85.1	4.8
Proposed	✓	✓	✓	**87.2**	**3.1**

## Data Availability

The original contributions presented in this study are included in the article; further inquiries can be directed to the corresponding author.
